# How to better communicate the exponential growth of infectious diseases

**DOI:** 10.1371/journal.pone.0242839

**Published:** 2020-12-09

**Authors:** Martin Schonger, Daniela Sele

**Affiliations:** 1 Lucerne School of Business, Hochschule Luzern, Lucerne, Switzerland; 2 Center for Law and Economics, Department of Humanities, Social and Political Sciences, ETH Zurich, Zurich, Switzerland; Middlesex University, UNITED KINGDOM

## Abstract

Exponential growth bias is the phenomenon whereby humans underestimate exponential growth. In the context of infectious diseases, this bias may lead to a failure to understand the magnitude of the benefit of non-pharmaceutical interventions. Communicating the same scenario in different ways (framing) has been found to have a large impact on people’s evaluations and behavior in the contexts of social behavior, risk taking and health care. We find that framing matters for people’s assessment of the benefits of non-pharmaceutical interventions. In two commonly used frames, most subjects in our experiment drastically underestimate the number of cases avoided by adopting non-pharmaceutical interventions. Framing growth in terms of doubling times rather than growth rates reduces the bias. When the scenario is framed in terms of time gained rather than cases avoided, the median subject assesses the benefit of non-pharmaceutical interventions correctly. These findings suggest changes that could be adopted to better communicate the exponential spread of infectious diseases.

## Introduction

Non-pharmaceutical interventions (NPIs), such as social distancing, wearing masks, quarantines, school closures or curtailing economic life, can slow down the spread of COVID-19 [1–3]. A nascent behavioral literature investigates how to improve the adherence to such NPIs, for example regarding social distancing [4–6], wearing masks [6–8], or self-isolating [5, 6, 9], for an overview see [10]. Absent options such as vaccines or treatments, behavioral adaptations may need to be sustained for many months. However, while their costs are directly felt, their benefit is abstract: they slow the prospective exponential growth of the infectious disease. Public support for and adherence to such measures depends on the correct assessment of their benefits by the public and/or by opinion and community leaders. Previous research [11–23] has shown that people underestimate exponential growth. For financial contexts, empirical research has shown that a biased perception of exponential growth causally affects real-world behavior [17], and in the infectious disease context, recent lab evidence suggests the same [22]. Teaching is a potential instrument by which to reduce the exponential growth bias, however, the evidence on its effectiveness is mixed. Sometimes teaching about exponential growth (bias) partially reduces bias [22, 23], while other times it has almost no impact [18, 21]. In line with this, being aware of exponential growth bias does not prevent subjects in our sample from drastically underestimating exponential growth.

The assessment of exponential disease spread and of the benefit of decreasing the underlying growth rate may depend on the way in which information is communicated. Indeed, regarding other decision problems in social behavior [24, 25], risk taking [26–29] and health care [30–33], different methods of communicating identical information about a scenario (i.e., different frames) have been found to alter people’s perception and evaluation of available choices. The aim of this study is to investigate whether different framing can facilitate the understanding of exponential growth. The results indicate that selecting different frames reduces exponential growth bias in the context of disease spread. Framing growth in terms of doubling times rather than growth rates and focusing on the time span until a certain threshold is reached drastically reduces bias. The results suggest ways in which the communication about disease spread and NPIs could be improved.

## Methods

The study received prior approval from the ETH Zurich ethics committee, EK 2020-N-36. Written consent was obtained from participants.

### Study design

In the experiment, we present subjects with a hypothetical scenario, in which a country can slow the spread of an infectious disease by adopting NPIs. Initially, the country has 974 cases of the infectious disease. Without mitigation measures, the number of cases grows by 26% a day, to about 1 million cases in 30 days. NPIs would reduce the daily growth rate to 9% a day, resulting in about 13,000 cases in 30 days. Looking at the benefit of the mitigation measures from a different perspective, the country would gain about 50 days until 1 million cases are reached.

Subjects are asked their beliefs concerning three questions: what the benefit of the mitigation measures (the NPIs) is (‘mitigation question’), how much the disease spreads if no mitigation measures are taken (‘high exponential growth question’) and by how much it spreads if mitigation measures are taken (‘low exponential growth question’).

The scenario and the three questions are presented to subjects in one of four frames. Each subject is randomly assigned to one frame. Frames vary along two dimensions, r vs. d and C vs. T. Dimension r vs. d concerns the way in which the growth of the disease is communicated: either in terms of the daily growth rate (r), or in the equivalent doubling time in days (d). Dimension C vs. T changes the perspective on the benefit of the mitigation measures: either in terms of the cases avoided within 30 days (C) or in terms of the time gained until 1 million cases are reached (T). This results in four frames, C-r, C-d, T-r, and T-d. Table 1 gives an overview of the four frames, and the complete questions are given under Procedure.

**Table 1:** Experimental stimuli in the four different frames.

**Frame C-r**	**Frame C-d**
Mitigation question:	Mitigation question:
Mitigation measures would reduce the daily growth rate from 26% to 9%. How many cases could be avoided in 30 days?	Mitigation measures would lengthen the doubling time from 3 days to 8 days. How many cases could be avoided in 30 days?
(986,330 cases)	(984,271 cases)
High [low] exponential growth question:	High [low] exponential growth question:
The number of infected people grows by 26% [9%] per day. How many people will be infected in 30 days?	The number of infected people doubles every 3 [8] days.
(999,253 [12,923] cases)	How many people will be infected in 30 days?
	(997,376 [13,105] cases)
**Frame T-r**	**Frame T-d**
Mitigation question:	Mitigation question:
Mitigation measures would reduce the daily growth rate from 26% to 9%. How much time could be gained until 1 million cases are reached?	Mitigation measures would lengthen the doubling time from 3 days to 8 days. How much time could be gained until 1 million cases are reached?
(50.46 days)	(50.02 days)
High [low] exponential growth question:	High [low] exponential growth question:
The number of infected people grows by 26% [9%] per day. How long will it take until 1,000,000 people are infected?	The number of infected people doubles every 3 [8] days. How long will it take until 1,000,000 people are infected?
(30.00 [80.46] days)	(30.01 [80.03] days)

For all frames, subjects are told that in a country, there are 974 cases of an infectious disease.

**Fig 1:**
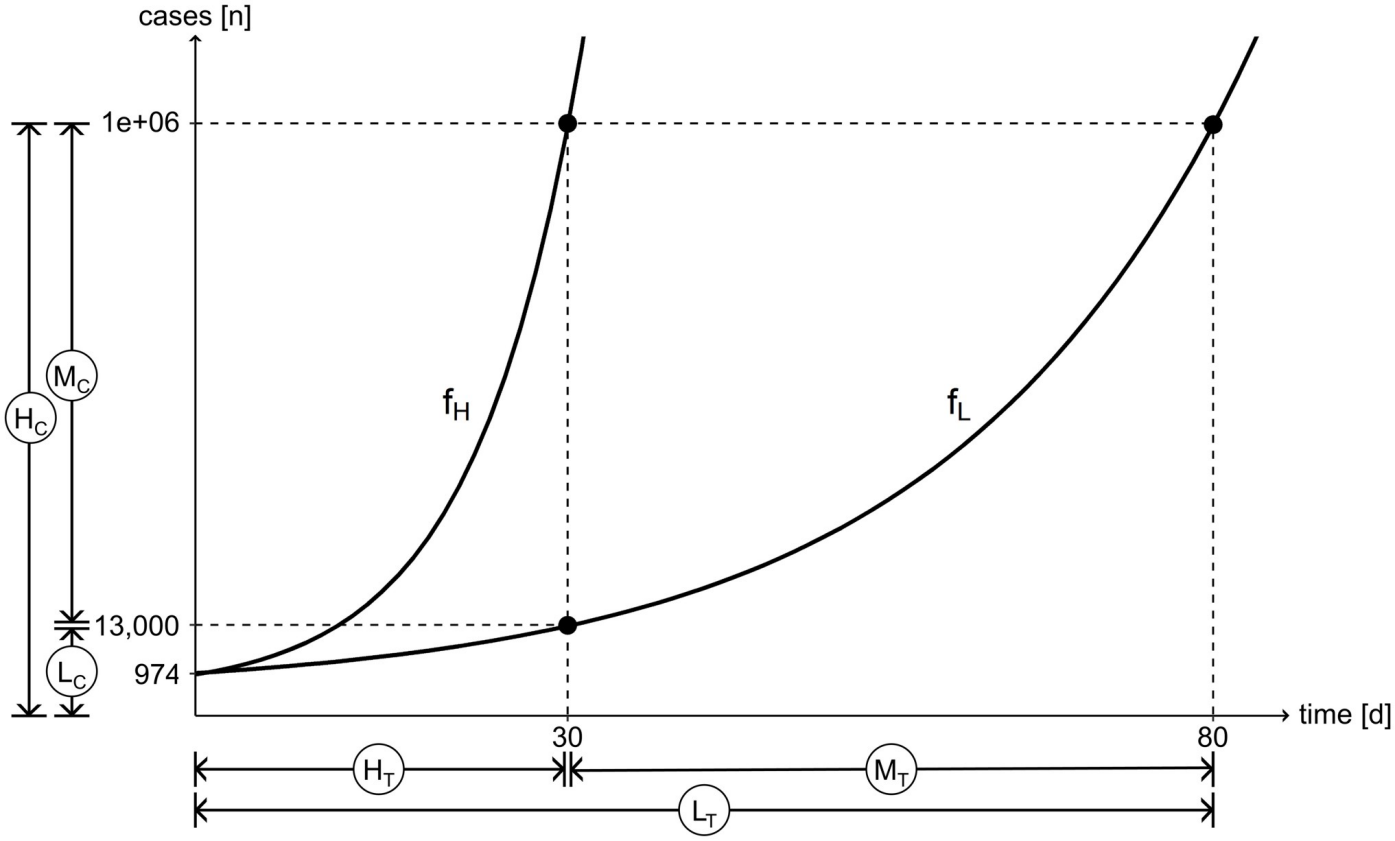
Schematic of questions. The correct answers to the mitigation, high exponential growth and low exponential growth questions for frames T-r and T-d are given by M_T_, H_T_ and L_T_, respectively. For frames C-r and C-d the answers are given by M_C_, H_C_ and L_C_, respectively. The high exponential growth function is *f*_H_ (26% per day/doubling time of 3 days), the low exponential growth function is *f*_L_ (9% per day/doubling time of 8 days). Not drawn to scale.

Fig 1 illustrates the underlying system. The parameters of the questions are set such that for the high exponential growth question the correct answer in frames C-r/C-d is about equal to the number of cases given in frames T-r/T-d, and, conversely, the correct answer in frames T-r/T-d is about equal to the amount of time given in frames C-r/C-d. The mitigation measures either reduce the number of cases in the country (C-r/C-d) or buy time for the country (T-r/T-d).

Hence, in all four frames subjects are given the same exponential function. However, in frames C-r/C-d, they are asked for cases as a function of time (an exponential problem) and in frames T-r/T-d, they are asked for time as a function of cases (a logarithmic problem). Anthropological evidence finds that people naturally employ logarithmic scales [34, 35], though this need not imply that people find logarithmic problems intuitive.

A subject exhibits exponential growth bias if they underestimate exponential growth. In frames C-r/C-d, this means underestimating the number of cases that result after a given time. In frames T-r/T-d, this means overestimating the amount of time until a given number of cases is reached. In line with this, we define mitigation bias as underestimating the benefit of decelerating the exponential spread of the disease. In frames C-r/C-d, this means underestimating the number of cases avoided due to the mitigation measures and in frames T-r/T-d, it means underestimating the number of days gained due to the measures.

Finally, the phenomenon, if not the term “exponential growth bias”, may already be known to subjects. To find out whether or not a subject is aware of the phenomenon, subjects are asked whether they believe others’ answers to the high exponential growth question are in general approximately correct, too low or too high.

### Procedure

Each subject sees three questions related to exponential processes, with each one presented on a separate screen: the mitigation question and the high and low exponential growth questions. The order of questions is randomized such that subjects either see the screen with the mitigation question first, or the two exponential growth questions first. The two exponential growth questions are further randomized within each other. Hence, within each frame, subjects are randomly assigned to one of four subgroups, where each subgroup sees the three questions in one of the following orders:

- Mitigation question, high exponential growth question, low exponential growth question;
- Mitigation question, low exponential growth question, high exponential growth question;
- High exponential growth question, low exponential growth question, mitigation question; and
- Low exponential growth question, high exponential growth question, mitigation question.

The following gives the full text of the mitigation, the high exponential growth, and the low growth exponential questions (translated from the original German). Brackets indicate the parts of the questions that differ across frames.

#### Mitigation question

In a country, 974 people have been infected so far. The number of infected people [grows by 26% daily] / [doubles every 3 days]. [The country wants to push back the moment when 1,000,000 people are infected as much as possible.]/[The country aims to have as few infected people as possible in 30 days.] Therefore, the adoption of measures, such as increased hand-washing and social distancing, is being discussed. With these measures, the number of infected people would [grow at merely 9% per day] / [double only every 8 days]. [How much time would the country gain with these measures until 1,000,000 people are infected?] / [How many infections could be avoided in the following 30 days with these measures?].

#### High exponential growth question

In a country, 974 people have been infected so far. The number of infected people [grows by 26% per day] / [doubles every 3 days]. [How long will it take until 1,000,000 people are infected in this country?] / [How many people will be infected in 30 days?]

#### Low exponential growth question

In a country, 974 people have been infected so far. The number of infected people [grows by 9% per day] / [doubles every 8 days]. [How long will it take until 1,000,000 people are infected in this country?] / [How many people will be infected in 30 days?]

In all these questions, the aim is to determine the subjects’ intuitive perception of exponential growth, rather than their skills using the internet or calculators. Hence, subjects are asked to refrain from using calculators or other tools. Subjects have no disadvantage from complying with this request, as the study offers a non-contingent participation fee. To avoid providing a hint about the magnitude of the correct answer, the answer box is free form entry, and no unit is specified. Subjects are instructed to always specify units in their answers wherever appropriate. Neutral examples on how to answer are used (for instance, do not answer 187, instead answer 1 m 87 cm or answer 1.87 m). Moreover, a remark on the screen clarifies that for the case count recoveries and deaths should also be included.

To find out whether a subject is aware of the phenomenon of exponential growth bias, the high exponential growth question of their frame is again shown to the subject. The subject is asked to indicate their belief of how other subjects answered this question on a 5-point-Likert scale, with the following options:

#### Frames C-r and C-d

The answers of most participants were far too low. / The answers of most participants were too low. / The answers of most participants were approximately correct. / The answers of most participants were too high. / The answers of most participants were far too high.

#### Frames T-r and T-d

Most participants indicated a timespan that was far too short. / Most participants indicated a timespan that was too short. / Most participants were approximately correct. / Most participants indicated a timespan that was too long. / Most participants indicated a timespan that was far too long.

### Subjects

The study was conducted online on 25 and 26 March 2020. During this time, all educational institutions and non-essential shops in Switzerland were closed due to SARS-CoV-2. Subjects were students in non-STEM fields at Swiss universities (n = 459). Subjects self-reported their age, gender and field of study, for these and further characteristics see S2 Table. Subjects are randomly assigned with equal probability to one of the four treatment groups/frames. The online experiment was implemented by the staff of the ETH Decision Sciences Laboratory (DeSciL). The authors had no contact with the subjects. Subjects were paid a non-contingent participation fee of CHF 10 (about USD 10 at the time of the study).

### Statistical methods

To test whether the fraction of biased subjects is larger in one frame than in another, we use Pearson’s chi-squared test, where the number of successes is defined as the number of subjects who give an answer that is below the true value. For the power calculation, assuming an effect size of 20%, and requiring *α* = 0.05 and 80% power, a minimum sample size of 97 subjects per group, or 388 subjects in total, is required. 495 subjects were invited to the study, and 459 subjects participated. This resulted in 116 subjects each in frames C-r, T-r and T-d, and 111 in frame C-d.

To test whether mitigation bias can be fully explained by exponential growth bias, we use a binomial test, where the number of successes is defined as the number of subjects who give an answer to the mitigation question that is larger than the difference between their answers to the exponential growth questions. To test whether the median answer differs between two frames, we use the Brown-Mood median test.

## Results and discussion

### Subjects underestimate the benefits of NPIs, but framing can eliminate the bias

**Fig 2:**
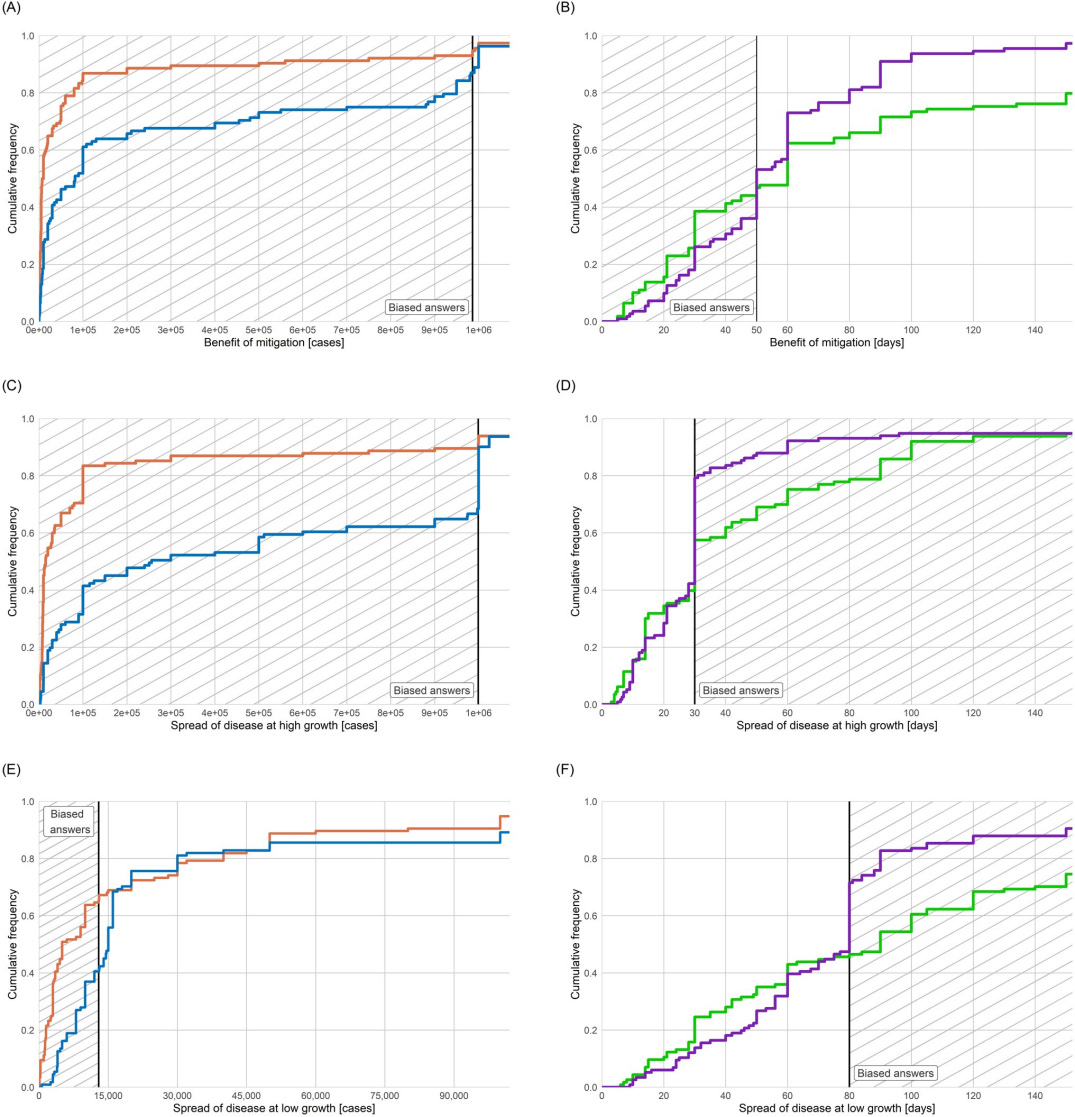
Effect of framing on bias. **A-B:** cumulative distribution function (CDF) of answers to the mitigation question, for A, frame C-r (orange line, n = 114)) and frame C-d (blue line, n = 102), B, frame T-r (green line, n = 109) and frame T-d (purple line, n = 111). **C-D:** CDF of answers to the high exponential growth question, C, frame C-r (n = 115) and C-d (n = 111), C, frame T-r (n = 109) and T-d (n = 111). **E-F:** CDF of answers to the low exponential growth question, E, frame C-r (n = 116) and C-d (n = 111), F, frame T-r (n = 113) and T-d (n = 116). Solid vertical line indicates correct answer. Hatched area indicates beliefs that reveal mitigation /exponential growth bias. Axes are capped.

Fig 2 gives the cumulative distribution functions of subjects’ answers, with the solid vertical lines indicating the correct answers and the shaded areas indicating beliefs with mitigation/exponential growth bias. For the mitigation question, in frames C-r/C-d (Fig 2A), NPIs avoid about 985,000 cases. In both frames, most subjects underestimate these benefits: 94% of subjects in frame C-r and 87% of subjects in frame C-d exhibit mitigation bias. These shares are not statistically significantly different at the 99%-level. The median answer is 8,600 cases avoided in frame C-r, and 82,000 cases avoided in frame C-d (for further quantiles, see extended data Table 1). Hence, the median answer in the frame using doubling times exhibits less bias than the median answer in the frame using growth rates (*p* < 10^−5^). For frames T-r/T-d, where mitigation measures buy the country about 50 days, consider Fig 2B: 44% of subjects in frame T-r and 36% of subjects in frame T-d believe that fewer days are gained (i.e., they exhibit mitigation bias). The fraction of subjects exhibiting mitigation bias is not statistically significantly different between the two frames at the 99%-level. The median assessment of the benefit of the mitigation measures is 60 days in frame T-r, and 50 days in frame T-d. The median answers are not statistically significantly different between the two frames at the 99%-level. Frames C-r/C-d employ the exponential perspective, and frames T-r/T-d employ the logarithmic perspective. Therefore, the median answers are not comparable, but one can compare these frames according to the fraction of biased subjects. In frame C-r, 50 percentage points more subjects are biased than in frame T-r (*p* < 10^−14^). In frame C-d, 51 percentage points more subjects are biased than in frame T-d (*p* < 10^−13^).

### Framing matters for the perception of the spread of infectious diseases

If the disease spreads at the high growth rate, there will be about 1 million cases in 30 days (Fig 2C). 90% of subjects in frame C-r and 67% of subjects in frame C-d underestimate this (i.e., they exhibit exponential growth bias). The median answer is 15,000 cases in frame C-r, and 256,000 cases in frame C-d. Framing the scenario using doubling times facilitates understanding: the share of subjects that exhibit the bias is lower (*p* < 10^−4^), and the median answer in that frame is closer to the correct amount (*p* < 10^−3^). Turning to frames T-r/T-d, the median answer in these frames coincides with the correct value of 30 days. 42% of subjects in frame T-r, and 21% of subjects in frame T-d believe it takes longer than that to reach 1 million cases (i.e., they exhibit exponential growth bias). Hence, the share of participants exhibiting exponential growth bias is lower when doubling times are used (*p* < 10^−3^). Comparing the exponential and logarithmic perspectives, in frame C-r, 48 percentage points more subjects are biased than in frame T-r (*p* < 10^−13^), and in frame C-d, 46 percentage points more subjects are biased than in frame T-d (*p* < 10^−11^).

If the disease spreads at the low growth rate, there will be about 13,000 cases after 30 days (Fig 2E). In frame C-r, the median answer is 5,000 cases, and 65% of subjects exhibit exponential growth bias. In frame C-d, which uses doubling times, the median answer is 15,000 cases and only a minority of subjects, 41%, exhibit exponential growth bias. The median answer in the frame using doubling times is closer to the correct amount than the median answer in the frame using growth rates. For frames T-r/T-d, the correct answer is about 80 days (Fig 2F). The median answer is 90 days in frame T-r, and 80 days in frame T-d. 54% of subjects in frame T-r, and 28% of subjects in frame T-d believe it takes longer to reach 1 million cases. Hence, we again find that the share of participants exhibiting exponential growth bias is lower when doubling times are used (*p* < 10^−4^). Comparing the exponential and logarithmic perspectives, in frame C-r, 11 percentage points more subjects are biased than in frame T-r (*p* = 0.056), and in frame C-d, 13 percentage points more subjects are biased than in frame T-d (*p* = 0.03).

### The frame using doubling time and the logarithmic perspective has the fewest biased subjects

The picture that emerges is that some methods of communicating exponential growth are better than others. Hence, the question arises which of the four frames least (most) effectively communicates exponential disease spread. The criterion we use to evaluate the effectiveness of a frame is the fraction of biased subjects. This criterion allows for pairwise comparison of any two frames. Table 2 presents these pairwise comparisons, giving the differences in the shares of biased subjects. For example, the first entry in Table 2, -7%, means that 7 percentage points fewer subjects give a biased answer to the mitigation question in frame C-d than in frame C-r. Below the percentage point difference, the 95%-confidence interval and the p-value are given.

**Table 2:** Difference in share of biased subjects across frames.

Mitigation question			
	C-d	T-r	T-d
C-r	-7% (-15%,1%) p = 0.07	-50% (-60%,-40%) p < 10^−14^	-58% (-68%, -48%) p < 10^−16^
C-d	-	-43% (-54%, -32%) p < 10^−10^	-51% (-62%, -40%) p < 10^−13^
T-r	-	-	-8% (-21%, 5%) p = 0.14
High exponential growth question			
	C-d	T-r	T-d
C-r	-24% (-34%, -13%) p < 10^−4^	-48% (-59%, -37%) p < 10^−13^	-70% (-79%, -61%) p < 10^−16^
C-d	-	-24% (-37%,-12%) p < 10^−3^	-46% (-57%, -35%) p < 10^−11^
T-r	-	-	-22% (-34%, -10%) p < 10^−3^
Low exponential growth question			
	C-d	T-r	T-d
C-r	-23% (-36%, -11%) p < 10^−3^	-11% (-24%, 1%) p = 0.056	-36% (-48%, -24%) p < 10^−7^
C-d	-	12% (-0.1%, 25%) p = 0.46	-13% (-25%, -0.1%) p = 0.03
T-r	-	-	-25% (-37%, -13%) p < 10^−4^

The 95% confidence intervals are given in parentheses. The p-value of the one-sided hypothesis test is given.

As one can see from the table, it turns out that the frame that least effectively communicates exponential growth is the same in all three questions: frame C-r. This frame communicates exponential growth in terms of the growth rate and asks for the number of cases. The share of biased subjects in this frame is statistically significantly higher than in all other frames, though only marginally so for frames T-r (*p* = 0.056) and C-d (*p* = 0.07). Regarding which frame most effectively communicates exponential growth, it turns out that one frame is most effective in all three questions: frame T-d, which communicates exponential growth in terms of doubling times and asks for an amount of time until a threshold of cases is reached. The share of biased subjects in this frame is statistically significantly lower than in all other frames, except for frame T-r (*p* = 0.14).

The four frames differ along two dimensions of communication, and it might be helpful to see these findings from this angle. First, compare communicating growth in terms of doubling times to communicating growth in terms of growth rates (i.e., compare frame C-d to C-r and T-d to T-r). In any comparison that differs only along this dimension, the share of biased subjects is smaller when doubling times are used. Second, in any comparison that differs only in whether the logarithmic perspective rather than the exponential perspective is prompted (i.e., compare frame C-d to T-d and C-r to T-r), the share of biased subjects is smaller with the logarithmic perspective. That is, fewer subjects exhibit mitigation bias and exponential growth bias when asked for time rather than cases.

### Mitigation bias is larger than exponential growth bias would predict

**Fig 3:**
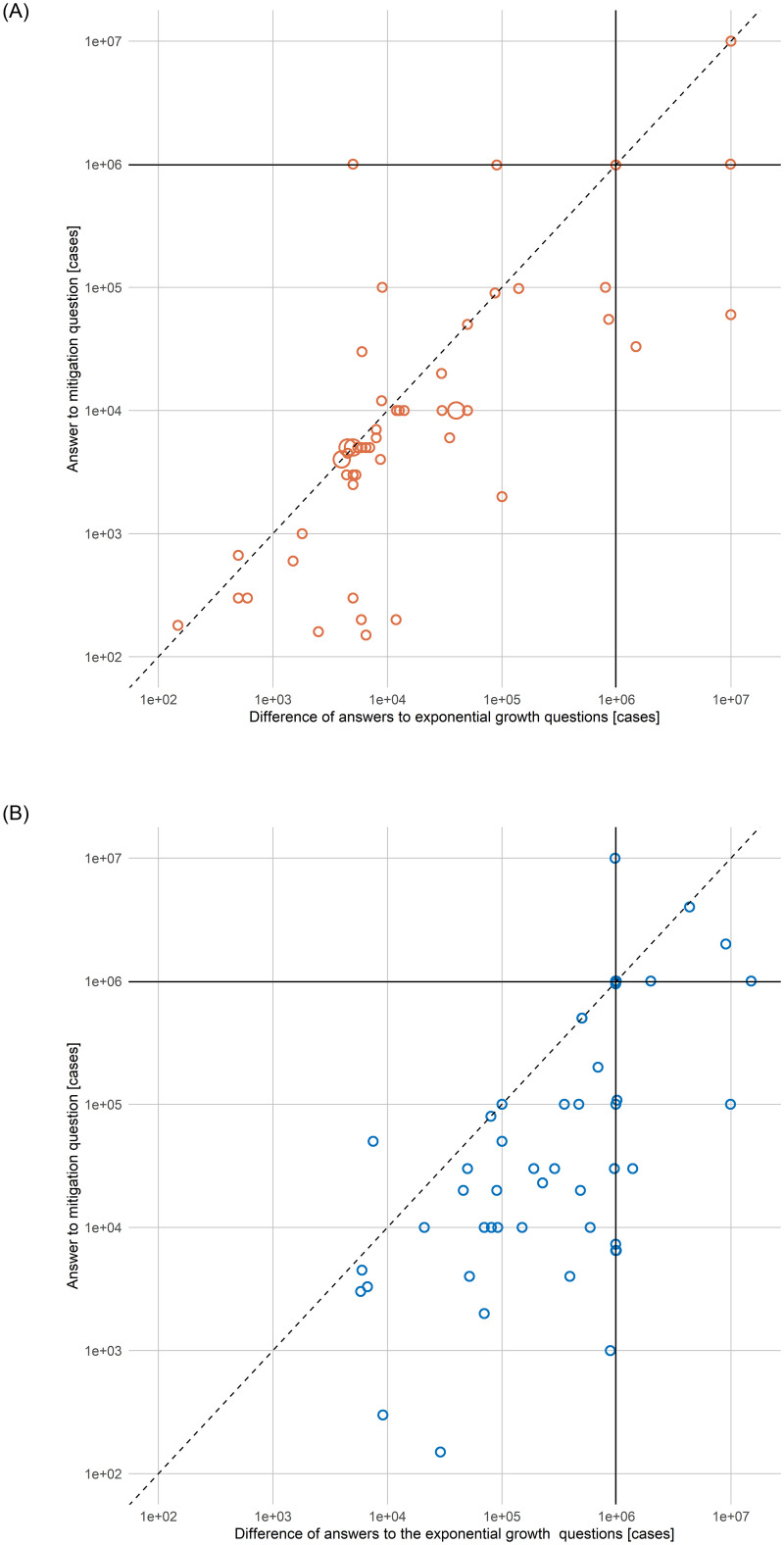
Mitigation bias and exponential growth bias. **A**: answers to the mitigation question plotted against the difference in answers to the exponential growth questions for frame C-r (n = 54). **B**: same plot for frame C-d (n = 50). Solid lines indicate the correct answer to the mitigation question, respectively, the difference between the correct answers to the exponential growth questions (about 1 million cases avoided). For observations on the dashed line, mitigation bias can be fully explained by exponential growth bias. Multiple identical answers are displayed by larger circles. Only subjects to whom the mitigation question was displayed prior to the exponential growth questions are included. Data points with non-positive values are excluded. One outlier in C-d is not shown.

In the following, we examine to what extent mitigation bias can be accounted for by exponential growth bias. The correct answer to the mitigation question is the difference between the correct answers to the two exponential growth questions. Hence, to relate the mitigation bias found in frames C-r and C-d to exponential growth bias, we compare a subject’s answer in the mitigation question to the difference between her answers to the high and low exponential growth questions (Fig 3). For this exercise, we restrict attention to positive data points and to those subjects to whom the mitigation question was displayed prior to the exponential growth questions. For 14% of subjects in frame C-r and 5% of subjects in frame C-d, the answer to the mitigation question is exactly equal to the difference in their answers to the exponential growth questions (for subjects who see the mitigation question after the exponential growth questions, this occurs more frequently; see extended data Fig 1). 66% of subjects in frame C-r and 76% of subjects in frame C-d give an answer to the mitigation question that is smaller than the difference in their answers to the exponential growth questions. Hence, for these subjects, mitigation bias is larger than one would expect based on their answers to the exponential growth questions. The null hypothesis that mitigation bias is not larger than exponential growth bias is rejected for both frame C-r (*p* < 0.05) and frame C-d (*p* < 10^−4^).

### Being aware of exponential growth bias does not prevent it

The phenomenon of exponential growth bias has been discussed for centuries [36, 37] and has received renewed attention in the media recently in the context of the coronavirus pandemic [38, 39]. To investigate subjects’ awareness of exponential growth bias, we ask subjects what they believe about other subjects’ answers to the high exponential growth question. 83% of subjects in frame C-r and 91% of subjects in frame C-d believe that others underestimate or strongly underestimate the number of cases. In both frames T-r and T-d, 66% of subjects believe that others overestimate or strongly overestimate the span of time. Hence, most subjects have an awareness of the phenomenon of exponential growth bias. Despite this awareness, subjects exhibit exponential growth bias and mitigation bias in the frames using an exponential perspective.

## Conclusion

In the commonly used frame of case growth and daily exponential growth rates, subjects in the experiment drastically underestimate the benefit of decreasing the growth rate of an infectious disease. Such biased beliefs about the exponential spread of COVID-19 decrease the willingness to adhere to NPIs [11]. We find that communicating exponential growth in terms of doubling times rather than growth rates decreases bias. Employing a logarithmic perspective (that is, asking for time gained rather than an exponential perspective, which asks for cases avoided), is even more effective at decreasing bias. The frame that combines doubling times with the logarithmic perspective fully eliminates mitigation bias. These findings suggest directions for changes that public health authorities could adopt in order to better communicate the benefits of NPIs and to increase adherence to them. Beyond public health, our findings may have applications in the regulation of the sale of financial products, retirement savings, education and the public understanding of exponential processes in the environment.

## Supporting information

S1 Fig Mitigation bias and exponential growth bias–subsample of subjects who see exponential growth questions first. A: Answers to the mitigation question plotted against the difference in answers to the exponential growth questions for frame C-r (n = 49). B: Same plot for frame C-d (n = 40). The solid line indicates the correct answer (about 1 million cases avoided). For observations on the dashed line, mitigation bias can be fully explained by exponential growth bias (28% in frame C-r, 23% in frame C-d). Multiple identical answers are displayed by larger crosses. Only subjects to whom the two exponential growth questions were displayed prior to the mitigation question are included. Data points with non-positive values are excluded.
(TIF)

S1 Table Question parameters and response quantiles. The table gives the parameters used in the three questions in the four different frames, the correct answers, and the 1st, 25th, 50th, 75th and the 99th percentiles of subjects’ responses.
(DOCX)

S2 Table Descriptive statistics of sample. The table gives descriptive statistics about the participants (n = 459). Table entries give n and percentage in brackets. Only students from non-STEM fields (according to the definition of the ETH Decision Sciences Laboratory) are invited to participate. Age is calculated as 2019 –birth year, which the participants self-report. Fields of study with a share > 10% are listed separately, all other fields are grouped under humanities, social sciences or other. Mathematical ability is self-reported on a 5-point-Likert-scale. Entries give number of participants, with share of sample in brackets. For age, quartiles are given.
(DOCX)
