# Supplementary figures and images for: How to better communicate the exponential growth of infectious diseases
Source: PLoS One. 2020 Dec 9;15(12):e0242839. doi: 10.1371/journal.pone.0242839 (PMC7725369; doi:10.1371/journal.pone.0242839)

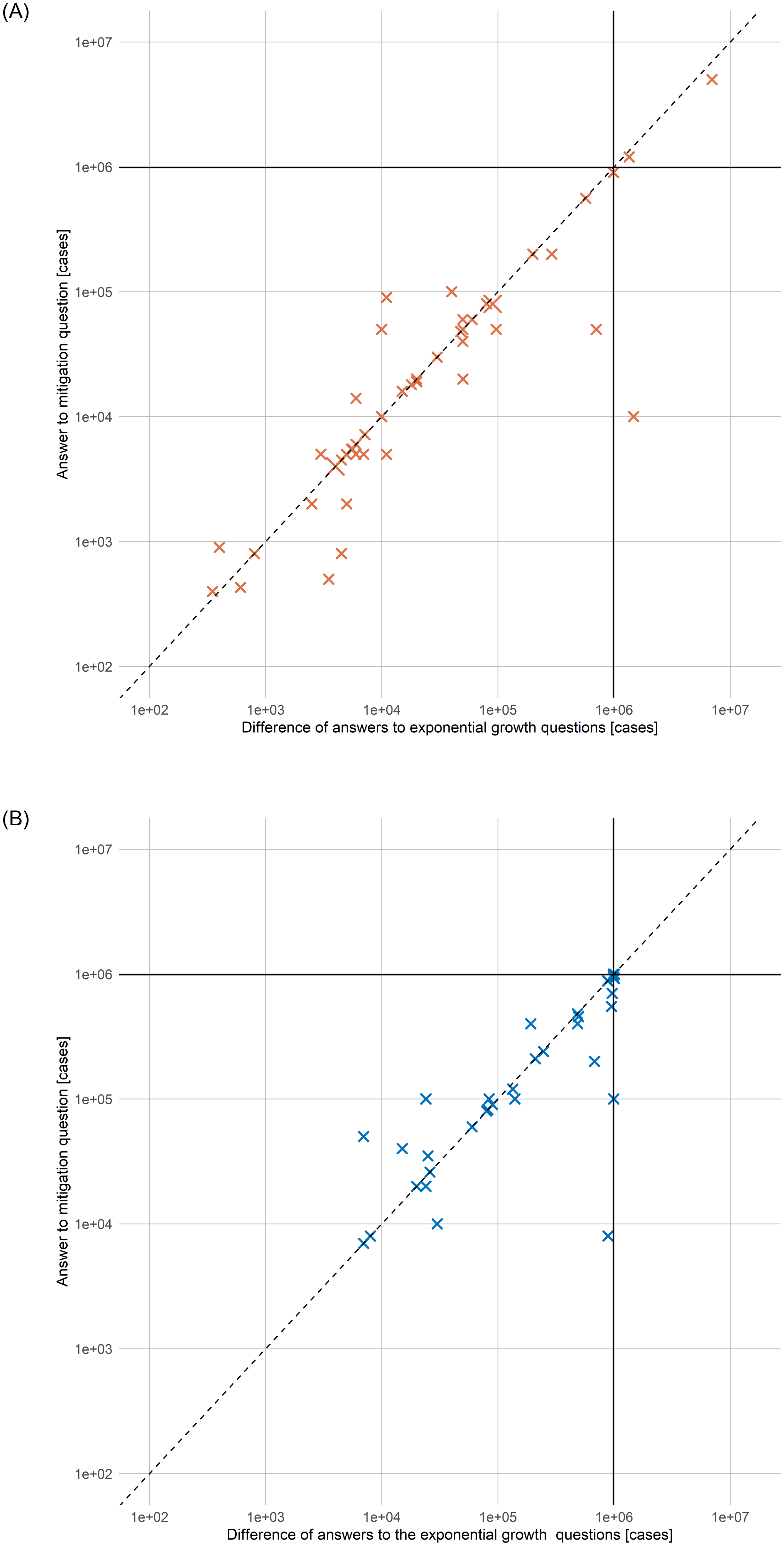

Supplement: S1 Fig — A: Answers to the mitigation question plotted against the difference in answers to the exponential growth questions for frame C-r (n = 49). B: Same plot for frame C-d (n = 40). The solid line indicates the correct answer (about 1 million cases avoided). For observations on the dashed line, mitigation bias can be fully explained by exponential growth bias (28% in frame C-r, 23% in frame C-d). Multiple identical answers are displayed by larger crosses. Only subjects to whom the two exponential growth questions were displayed prior to the mitigation question are included. Data points with non-positive values are excluded. (TIF) [file pone.0242839.s001.tif]
