# Supplementary material for: How to better communicate the exponential growth of infectious diseases
Source: PLoS One. 2020 Dec 9;15(12):e0242839. doi: 10.1371/journal.pone.0242839 (PMC7725369; doi:10.1371/journal.pone.0242839)
Supplement: S1 Table — The table gives the parameters used in the three questions in the four different frames, the correct answers, and the 1st, 25th, 50th, 75th and the 99th percentiles of subjects’ responses. (DOCX) [file pone.0242839.s002.docx]

| **S1 Table. Question parameters and response quantiles** | | | | | | | | | | | | | | |
| --- | --- | --- | --- | --- | --- | --- | --- | --- | --- | --- | --- | --- | --- | --- |
| Question/ Frame | Question parameters | | | | |  | Correct answer | |  | Response quantiles | | | | |
|  | Initial cases | Growth rate | Doubling time [days] | Period [days] | Final cases |  | Cases | Days |  | 1% | 25% | 50% | 75% | 99% |
| Mitigation question | | | | | | | | | | | | | | |
| C-r | 974 | 26% / 9% | - | 30 | - |  | 986,330 | - |  | 18.69 | 4,000 | 8,600 | 50,000 | 4,506,000 |
| C-d | 974 | - | 3 / 8 | 30 | - |  | 984,271 | - |  | 349 | 10,000 | 82,000 | 745,000 | 9,542,800 |
| T-r | 974 | 26% / 9% | - | - | 1,000,000 |  | - | 50.46 |  | 5.16 | 28 | 60 | 120 | 1,087.4 |
| T-d | 974 | - | 3 / 8 | - | 1,000,000 |  | - | 50.02 |  | 8.1 | 30 | 50 | 70 | 180 |
| High exponential growth question | | | | | | | | | | | | | | |
| C-r | 974 | 26% | - | 30 | - |  | 999,253 | - |  | 814 | 8,000 | 15,000 | 100,000 | 10,000,000 |
| C-d | 974 | - | 3 | 30 | - |  | 997,376 | - |  | 3,090 | 42,500 | 256,000 | 1,000,000 | 14,500,000 |
| T-r | 974 | 26% | - | - | 1,000,000 |  | - | 30.00 |  | 4 | 14 | 30 | 60 | 3,519.6 |
| T-d | 974 | - | 3 | - | 1,000,000 |  | - | 30.01 |  | 6.1 | 20 | 30 | 30 | 405.5 |
| Low growth exponential growth question | | | | | | | | | | | | | | |
| C-r | 974 | 9% | - | 30 | - |  | 12,923 | - |  | 93,2 | 2,907.5 | 5,000 | 30,000 | 10,000,000 |
| C-d | 974 | - | 8 | 30 | - |  | 13,105 | - |  | 2,550 | 8,000 | 15,000 | 20,000 | 1,000,000 |
| T-r | 974 | 9% | - | - | 1,000,000 |  | - | 80.46 |  | 7.13 | 35 | 90 | 172.5 | 1,400.2 |
| T-d | 974 | - | 8 | - | 1,000,000 |  | - | 80.03 |  | 10 | 50 | 80 | 88 | 802.75 |
