# Supplementary material for: How to better communicate the exponential growth of infectious diseases
Source: PLoS One. 2020 Dec 9;15(12):e0242839. doi: 10.1371/journal.pone.0242839 (PMC7725369; doi:10.1371/journal.pone.0242839)
Supplement: S2 Table — The table gives descriptive statistics about the participants (n = 459). Table entries give n and percentage in brackets. Only students from non-STEM fields (according to the definition of the ETH Decision Sciences Laboratory) are invited to participate. Age is calculated as 2019 –birth year, which the participants self-report. Fields of study with a share > 10% are listed separately, all other fields are grouped under humanities, social sciences or other. Mathematical ability is self-reported on a 5-point-Likert-scale. Entries give number of participants, with share of sample in brackets. For age, quartiles are given. (DOCX) [file pone.0242839.s003.docx]

**S2 Table. Descriptive statistics of sample.**

| Age | | | | | | | |
| --- | --- | --- | --- | --- | --- | --- | --- |
|  | 1^st^ quartile | Median | 3^rd^ quartile |  |  |  |  |
|  | 21 | 22 | 25 |  |  |  |  |
| Gender | | | | | | | |
|  | Female | Male | Declined to answer |  |  |  |  |
|  | 332 (72%) | 115 (25%) | 12 (3%) |  |  |  |  |
| University | | | | | | | |
|  | U of Zurich | ETH Zurich | Other school | Not enrolled |  |  |  |
|  | 340 (74%) | 85 (19%) | 31 (7%) | 3 (1%) |  |  |  |
| Level of studies | | | | | | | |
|  | Bachelor | Master | PhD | Other level | Not enrolled |  |  |
|  | 314 (68%) | 126 (27%) | 11 (2%) | 5 (1%) | 3 (1%) |  |  |
| Subject of studies | | | | | | | |
|  | Medicine | Law | Architecture | Social Sci. | Humanities | Other | No answer |
|  | 111 (24%) | 68 (15%) | 51 (11%) | 73 (16%) | 54 (12%) | 98 (21%) | 4 (1%) |
| Self-reported mathematical ability | | | | | | | |
|  | Very bad | Bad | Average | Good | Very good | Declined to answer |  |
|  | 20 (4%) | 85 (19%) | 211 (46%) | 123 (27%) | 16 (3%) | 4 (1%) |  |

The table gives descriptive statistics about the participants (n=459). Table entries give n and percentage in brackets. Only students from non-STEM fields (according to the definition of the ETH Decision Sciences Laboratory) are invited to participate. Age is calculated as 2019 – birth year, which the participants self-report. Fields of study with a share > 10% are listed separately, all other fields are grouped under humanities, social sciences or other. Mathematical ability is self-reported on a 5-point-Likert-scale. Entries give number of participants, with share of sample in brackets. For age, quartiles are given.
